# Variations in dietary fibre and added sugar intake across global, regional and national levels: implications for non-communicable diseases

**DOI:** 10.1017/S1368980026103188

**Published:** 2026-07-21

**Authors:** Maab Mohammed Alballa, Xinran Feng, Ahmed Mohamed Hassan, Xin Qi, Xin Liu

**Affiliations:** 1 Department of Epidemiology and Biostatistics, School of Public Health, Global Health Institute, Xi’an Jiaotong University, China; 2 Key Laboratory of Environment and Genes Related to Diseases Ministry of Education, Xi’an Jiaotong University, Xi’an, 710061, China

**Keywords:** Dietary fibre, Added sugar, Non-communicable diseases, Global dietary database, Dietary trends

## Abstract

**Objective:**

To examine global, regional and national trends in dietary fibre and added sugar consumption and to assess their implications for non-communicable disease (NCD) prevalence.

**Design:**

This study used a longitudinal ecological design. Dietary fibre (1990–2018) and added sugar (1990–2020) intake data were obtained from the Global Dietary Database (GDD). Intakes were analysed across GDD regions, age groups, education levels and urbanisation status. Subsequently, mixed-effects models were used to assess the association between NCD prevalence and intakes of fibre, added sugar and the fibre-to-added-sugar ratio, adjusting for socio-demographic index (SDI), national income and urbanisation.

**Setting:**

The GDD covers dietary intake estimates for 185 countries and territories.

**Results:**

Globally, median dietary fibre intake rose from 14·8 to 21·6 g/d, while added sugar intake increased from 7·7 % to 10·5 % of total energy intake. Fibre intake increased in all regions except high-income countries, where it declined from 21·5 to 16·4 g/d. In contrast, added sugar intake increased in all regions except Latin America and the Caribbean (15·6 % to 13·3 % of total energy). Intake patterns were associated with education and urbanisation levels, with higher added sugar intake observed in countries with a higher SDI (*r* = 0·195, *P* = 0·008). The association between dietary fibre-to-added-sugar ratio was not significantly associated with NCD prevalence (*β* = –10·15, *P* = 0·067).

**Conclusions:**

This study highlights global disparities in dietary fibre and added sugar consumption, driven by regional, educational and urbanisation factors. These findings underscore the need for targeted interventions to improve nutrition and reduce the NCD burden.

A healthy diet is the cornerstone of human well-being^(1)^. There may be variations in the intake of healthy and unhealthy dietary elements based on factors such as sex, ethnicity, culture and socioeconomic status^(2)^. Dietary fibre is an essential component of healthy diets, which is primarily composed of plant-sourced carbohydrates that are indigestible in the upper intestine and considered a crucial nutrition and energy source for the colonic microbiota in the large intestine^(3)^.

Globally, non-communicable diseases (NCD) remain a leading cause of morbidity and mortality. In 2021, they were responsible for an estimated 7·3 trillion cases worldwide, 43·8 million deaths and 1·73 billion disability-adjusted life years. Age-standardised rates per 100 000 population were 91 034 for prevalence, 529·7 for deaths and 20 783 for disability-adjusted life years, reflecting modest changes in prevalence (–0·1 %) but notable declines in mortality (–27·9 %) and disability-adjusted life years (–19·4 %) compared with 1990^(4)^. Observational studies have shown that increased fibre consumption is strongly associated with a lower risk of acquiring various chronic diseases, including type 2 diabetes, CVD, stroke and colorectal cancer, as well as lower cardiovascular and all-cause mortality^(5)^. Several studies highlight how dietary fibre intake contributes to improved cardiometabolic health, with the gut microbiota contributing to the positive outcomes^(6–8)^. Recommendations for adequate dietary fibre consumption vary by world region and age group, while adults are generally advised to at least consume 25–30 g per day^(9)^. As concerned, there is no geographic region that meets the minimal fibre consumption considered to be needed for good health worldwide^(3)^.

Conversely, the global consumption of sugar has resulted in a range of health concerns^(10)^. Added sugars refer to mono- and disaccharides that are incorporated into foods during their processing and preparation. Excessive intake of added sugars has been recognised as a risk factor contributing to the global disease burden, primarily NCD, obesity and dental caries^(11,12)^. The WHO recommends that sugar should account for less than 10 % of total caloric intake, while the EAT-Lancet suggests a maximum of 31 g of all types of sweeteners per day for one person^(13)^. Nevertheless, the overall consumption of free and added sugars continues to exceed the recommended guidelines^(14)^.

A few studies have examined dietary fibre and added sugar consumption over time, with only one reported the global trends^(9)^, another used sales data instead of intake data^(11)^ and others focused on specific populations or geographic areas^(15–19)^. This highlights the need for a comprehensive evaluation of the trends and further investigation into the potential factors influencing their intake. While a previous study using the Global Dietary Database (GDD) has used multivariate clustering in a largely exploratory manner to identify dietary patterns across countries^(20)^, our approach will focus specifically on dietary fibre and added sugar. This will allow us to examine their trends over time and their associations with NCD while controlling for potential confounders. By applying a more targeted, regression-based approach, this study was designed to link intake trends to health outcomes.

Effectively combating diet-related diseases worldwide requires understanding global food consumption patterns and trends, informing the development of dietary guidelines and the implementation of effective strategies. For this reason, we aimed to utilise the GDD^(21)^ to describe the global, regional and national trends of dietary fibre (1990–2018) and added sugar (1990–2020) among different age groups, levels of educational and degrees of urbanisation and their relationships with NCD.

## Methods

### Study design

This study employs a longitudinal ecological design to describe global, regional and national trends of dietary fibre intake (1990–2018) and added sugar intake (1990–2020) and their implications for NCD across 185 countries.

### Data source and extraction

In this study, the dietary data were obtained from the GDD, which is affiliated with the Global Nutrition and Policy Consortium at Tufts University (see online supplementary material, Supplemental Table S1 for variable definitions). The GDD provides detailed and comparable dietary intake estimates of nutrients and major foods, encompassing a total of 185 countries and territories. Standardised procedures for data collection, as well as subsequent data extraction and modeling, have been reported^(21)^. The GDD utilises data on individually based dietary intakes from existing surveys at the national and subnational levels. In cases where such surveys were unavailable for a country, individual-level surveys from large cohorts, the WHO Global Infobase and the WHO Stepwise Approach to Surveillance database were utilised. No eligible dietary surveys were identified for nineteen of 207 countries worldwide, including Afghanistan, Andorra, Bermuda, Cuba, Democratic People’s Republic of Korea, Djibouti, Equatorial Guinea, French Polynesia, Guadeloupe, Guinea-Bissau, Hong Kong, Macau, Martinique, Netherlands Antilles, Nicaragua, Palau, Réunion, Somalia and Turkmenistan. Estimates for these countries rely entirely on modelled data and are therefore subject to the greatest uncertainty, despite collectively representing approximately 1 % of the global population in 2015^(21)^.

According to the GDD, the world is categorised into seven regions: Latin America and the Caribbean, South Asia, the Middle East & North Africa, East and Southeast Asia, the Former Soviet Union, High-income Countries and Sub-Saharan Africa (see online supplementary material, Supplemental Table S2). The GDD offers estimates of dietary intake that are jointly stratified by age (0–1 year, 1–2 years, 2–5 years and then by increments of 5 years to age 97·5), sex (male *v*. female), education level (low, medium and high) and residence (urban *v*. rural). GDD provides data points for the years 1990, 1995, 2000, 2005, 2010, 2015, 2018 and 2020; however, dietary fibre data are only available up to 2018.

For this study, we acquired estimates of dietary fibre intake (g/d) from 1990 to 2018 and added sugar intake (% of total kcal/d) from 1990 to 2020 for adults aged ≥ 20 years across 185 countries. The dietary data were extracted at various levels and stratified by the GDD seven regions, age groups, education level and urbanisation status. Eight countries had missing dietary fibre and added sugar intake data for some years at age 95+. For these missing values, we substituted data from the 90–94 age group, assuming intake is similar across these older ages and to maintain complete country-year coverage. Age-specific population weights for each country and year were obtained from the United Nations Population Division. Based on above, a weighted average method was applied to estimate the dietary intake of adults aged 20 and older.

The prevalence rate data for NCD, expressed as total cases per 100 000 population for each country, was sourced from the Global Burden of Disease (GBD)^(22)^ from 1990 to 2018. The GBD 2019 conducted a detailed annual analysis of 369 diseases and injuries across 204 countries and territories, grouped into twenty-one GBD regions, covering a 30-year period. In this study, NCD were defined as the GBD 2019 Level 1 aggregate, which refer to the aggregate of all NCD included in the GBD 2019 database. The term encompasses various health conditions, including CVD, neoplasms, chronic respiratory diseases, diabetes and kidney diseases, digestive diseases and other NCD categories. The socio-demographic index (SDI), obtained from the GBD, is an essential indicator of social development. This composite measure takes into account income per capita, the average education level of individuals aged 15 and older and the total fertility rate for those under 25. The SDI varies from 0 to 1, representing a range from the least developed to the most developed.

Furthermore, covariate data related to socio-economic factors were sourced from the World Development Indicators, which provides a diverse array of indicators that can impact health outcomes. The study utilised the following indicators: Gross National Income, Tertiary School Enrollment (% of Gross) and Rural Population (% of Total Population). Missing data were present for Gross National Income (5 %) and school enrollment (34 %). Linear interpolation was used to address missing values within countries when data were unavailable for some years, as previously described^(23)^. For the eight countries with no school enrollment data across all years, values were replaced with data from country with the closest SDI, as SDI includes educational attainment and supports comparability across similar countries. The NCD prevalence data, SDI data and indicators data were subsequently matched by year and country with the GDD data. Together, these databases provide a robust foundation for understanding the relationships between dietary fibre intake, added sugar intake and NCD.

### Data analysis

This study examined trends in dietary fibre and added sugar intake over the past three decades, highlighting the differences in intake across various age groups by world region. These trends and age-related differences were illustrated through line graphs. Absolute differences in intake were calculated at the stratum level and aggregated to determine global and regional levels. This allowed for median comparisons between urban/rural areas and high/low education levels (≤ 6 years *v*. > 12 years of formal education).

National-level dietary fibre and added sugar consumption patterns were visualised using intake distribution maps created in ArcMap (version 10.8). A Pearson correlation test was used to assess the correlation between dietary fibre intake and SDI, and between added sugar intake and SDI, at the country level.

The associations of dietary fibre intake and added sugar intake with NCD were evaluated using linear mixed models. In this analysis, two models were employed.

Model 1 was adjusted for year and *SDI* as fixed effects:

$$NCDprevalence_{it}=\beta_{0}+\beta_{1}\cdot Exposure_{it}+\underset{k}{\sum }\gamma_{k}\cdot Year_{k,it}+\beta_{2}\cdot SDI_{it}+u_{i}+\epsilon_{it}$$

$NCDprevalence_{it}$ is the number of NCD cases per 100 000 population in country i at year t (unit of analysis: country-year). $Exposure_{it}$ is the dietary exposure variable. *Year* is included as a categorical variable. $SDI_{it}$ is the socio-demographic index. $u_{i}$ = random intercept for country. $\varepsilon_{it}$ = residual error.

Model 2 further included *GNI*, rural population and school enrollment as fixed effects:

$$NCDprevalence_{it}=\beta_{0}+\beta_{1}\cdot Exposure_{it}+\underset{k}{\sum }\gamma_{k}\cdot Year_{k,it}+\beta_{2}\cdot SDI_{it}+\beta_{3}\cdot GNI_{it}+\beta_{4}\cdot RuralPop_{it}+\beta_{5}\cdot SchoolEnroll_{it}+u_{i}+\epsilon_{it}$$

GNI= Gross National Income. RuralPop = Rural population. SchoolEnroll= School enrollment.

In both models, country was included as a random intercept to account for between-country heterogeneity and within-country clustering over time. Countries were included as random intercepts for three reasons: (1) the data have a hierarchical structure, with repeated observations nested within countries; (2) the number of countries (> 30) is sufficient for reliable variance estimation and (3) our focus is on overall trends rather than country-specific intercepts. All other variables were treated as fixed effects because they represent either primary exposures or potential confounders that could influence the relationship between diet and NCD prevalence. Controlling for these covariates allows us to estimate the independent effect of dietary exposures on NCD outcomes.

We also explored the relative ratio of dietary fibre intake to added sugar intake as potential factors influencing the prevalence of NCD. Our goal was to understand how these dietary components may contribute to the occurrence of NCD. The relative ratio was calculated as dietary fibre intake divided by added sugar intake. Using the same two-model structure as for dietary fibre and added sugar, we examined the fibre-to-added-sugar ratio in separate models, with the ratio included as a fixed effect. Statistical analyses were performed in R (version 4.1.2). Linear mixed-effects models were fitted using lmer () from the lmerTest package in R, with *P* values < 0·05 considered statistically significant. Detailed diagnostic models are presented in the online supplementary material, Supplemental Material (Figure S1, S2, S3).

## Results

### Trends in global, regional and national dietary fibre (1990–2018) and added sugar (1990–2020) intake

Globally, dietary fibre and added sugar intake have been increased over the years. Median dietary fibre intake increased from 14·8 g/d (95% UI 13·8, 15·8) to 21·6 g/d (95% UI 20·6, 22·6). Meanwhile, added sugar intake increased from 7·7 % of total kcal/d (95% UI 5·5, 10·8) to 10·5 % of total kcal/d (95% UI 7·4, 13·4).

**Figure 1.**
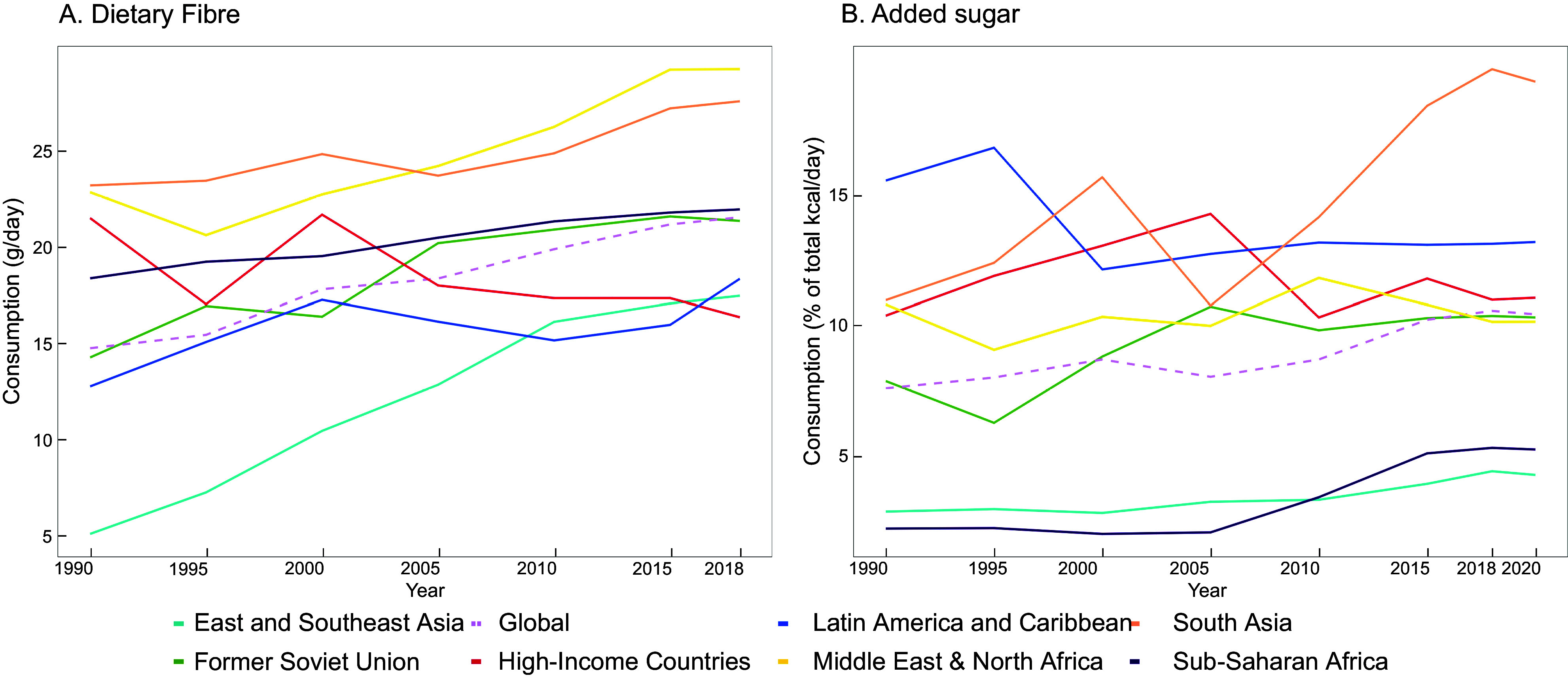
Figure 1 long description. Global and regional consumption trends of (a) dietary fibre (1990–2018) and (b) added sugar (1990–2020).

Regionally, dietary fibre intake increased in all regions between 1990 and 2018, except in high-income countries, which experienced a decrease from 21·5 g/d (95% UI: 20·8, 22·3) to 16·4 g/d (95% UI: 15·9, 16·8). The Middle East and North Africa had the highest 2018 fibre intake (29·2 g/d, 95% UI 26·5, 32·4), followed by South Asia (27·6 g/d, 95% UI 26·2, 29·1) and Sub-Saharan Africa (22 g/d, 95% UI 18, 26·5) (Figure 1(a)). Regarding added sugar, all regions showed an increase in intake between 1990 and 2020, except for Latin America and Caribbean experienced a decrease from 15·6 % of total kcal/d (95% UI 13·1, 18·3) to 13·3 % of total kcal/d (95% UI 10·8, 16·1). South Asia reported the highest added sugar intake in 2020 (19·4% of total kcal/d, 95% UI 9·9, 28·6), while East and Southeast Asia had the lowest (4·3%, 95% UI 2·4, 7·4) (Figure 1(b)).

**Figure 2.**
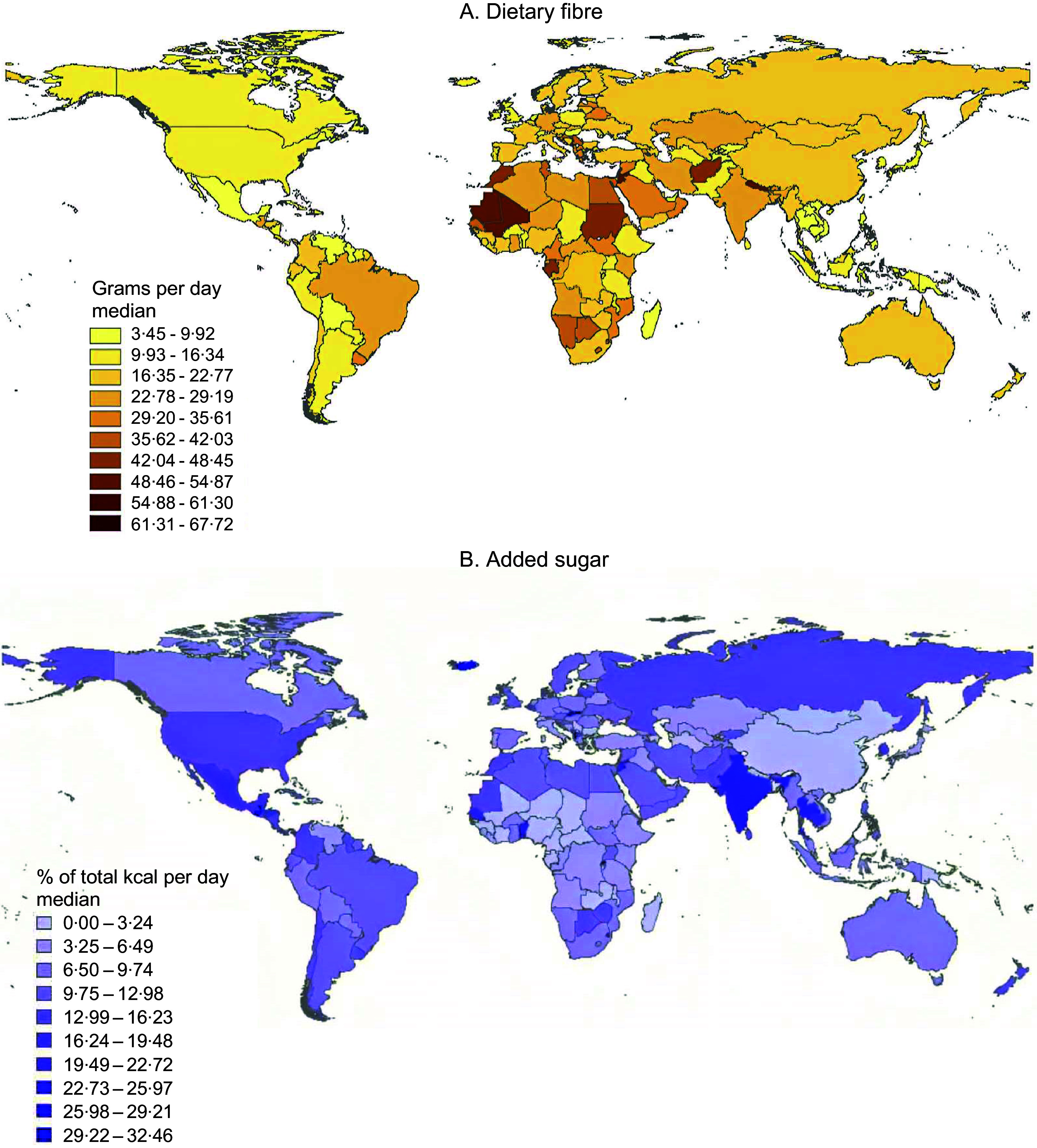
Figure 2 long description. National consumption of (a) dietary fibre (2018) and (b) added sugar (2020).

In 2018, median dietary fibre intake was the highest in Palestine (67·7 g/d), followed by Maldives (62·4 g/d), Montenegro (60·4 g/d), Cape Verde (58·8 g/d) and Mauritius (54·7 g/d). Conversely, the lowest intakes were observed in Guinea (3·49 g/d), Solomon Islands (3·5 g/d), Madagascar (5·5 g/d), Cambodia (5·6 g/d) and Vietnam (6·4 g/d) (Figure 2(a)). In 2020, Albania (32·4 %) ranked the highest intake of median added sugar (% of total kcal/d), followed by Rwanda (32·2 %), Guatemala (30·8 %) and Slovakia (30·2 %). The lowest intakes were observed in Solomon Islands (0·021 %), Turkmenistan (0·036 %), Papua New Guinea (2·13 × 10⁻^66^%) and Niger (18·3 × 10⁻^65^%) (Figure 2(b)).

### Age-specific dietary fibre (2018) and added sugar (2020) intake

**Figure 3.**
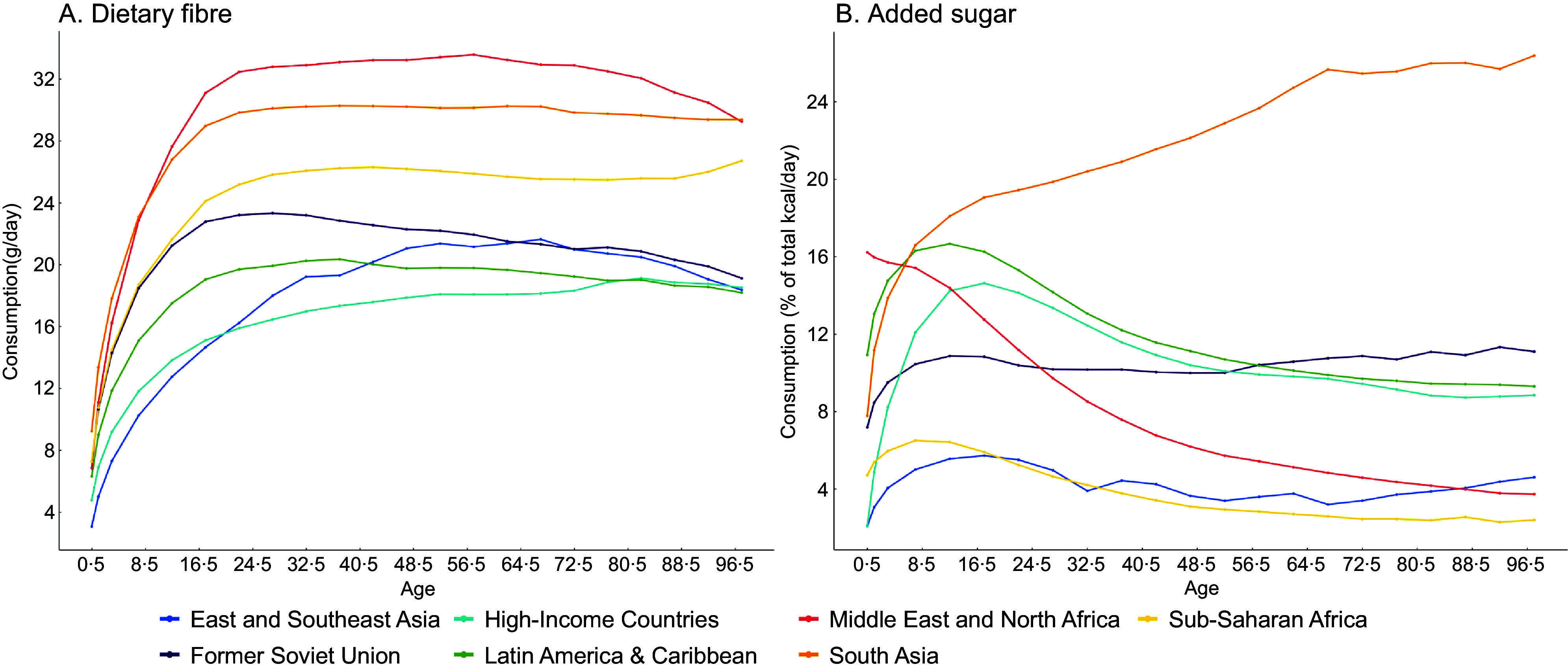
Figure 3 long description. Age-specific regional consumption of (a) dietary fibre (2018) and (b) added sugar (2020).

In the Middle East and North Africa, dietary fibre intake was the highest across almost all age groups compared with other regions, peaking at a median intake of 33·6 g/d among adults aged 55–59 years (Figure 3(a)). Dietary fibre intake increased with age in East & Southeast Asia, High-Income Countries and Sub-Saharan Africa. In contrast, dietary fibre intake decreased with age in the Middle East and North Africa, the Former Soviet Union and Latin America and the Caribbean, with the magnitude of these changes varying by region. Meanwhile, in South Asia, dietary fibre intake showed minimal variation between adults and elderly.

South Asia had the highest added sugar intake across all age groups compared with any other region, with adults aged 20–24 years having a median intake of 19·4 % of total daily caloric intake, while those aged 95+ years had a median intake of 26·4 %. In the Former Soviet Union, added sugar intake was slightly higher in older aged 95 + *v*. adults. In contrast, all other regions showed a decrease in added sugar intake among older individuals compared with younger adults (Figure 3(b)).

### Dietary fibre and added sugar intake by education level and place of residence

Globally, dietary fibre intake was consistently higher among the lower educated from 1990 to 2018. Added sugar consumption, initially higher among the higher educated, shifted towards the lower educated between 2015 and 2020. Both dietary fibre and added sugar intake, initially concentrated in urban areas, later transitioned towards non-urban areas.

**Figure 4.**
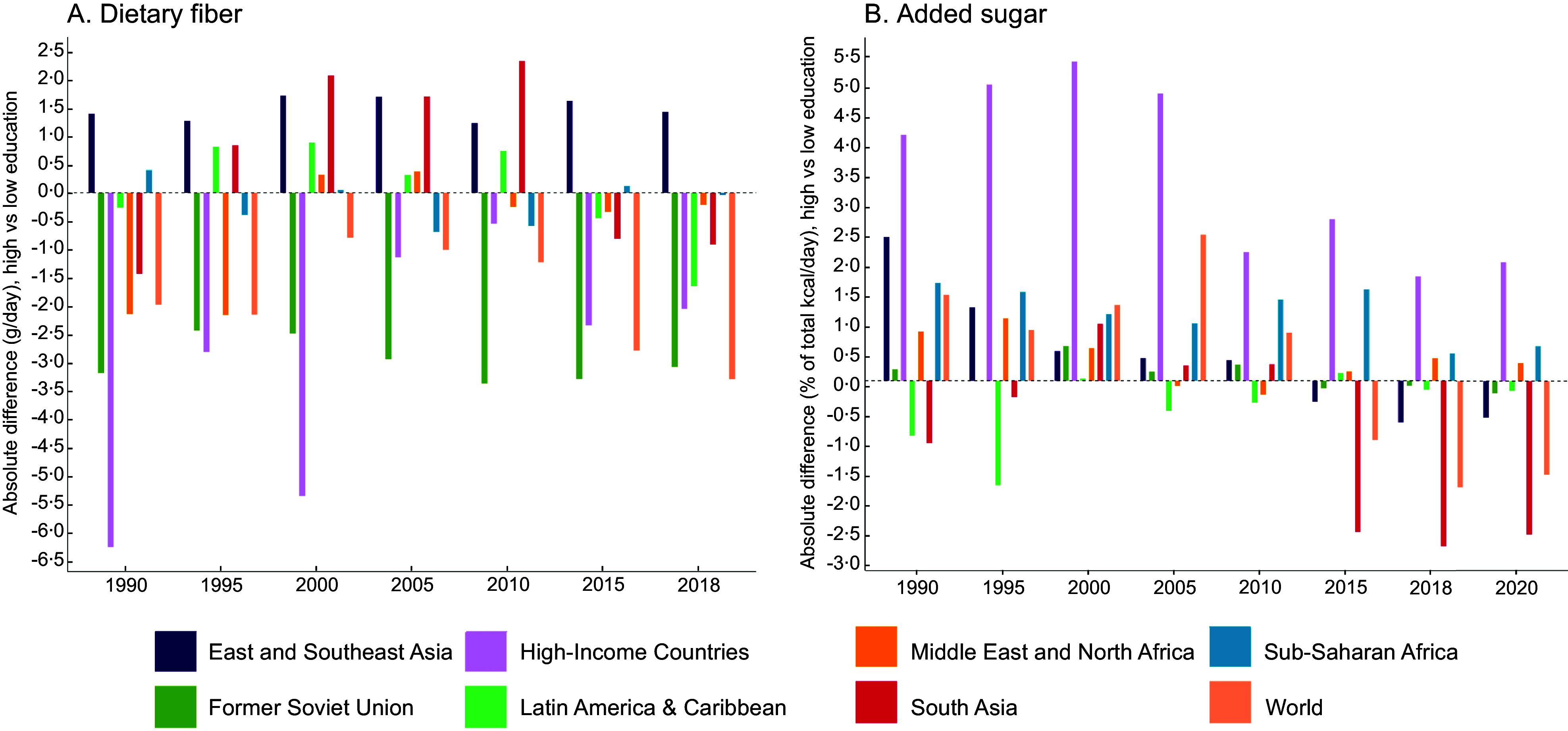
Figure 4 long description. Median difference in absolute dietary intake by education level and region. (a) Dietary fibre intake (1990–2018). (b) Added sugar intake (1990–2020). Education level is defined as ≤ 6 years *v*. > 12 years of formal education. A positive value indicates that the median intake in the high education group is higher, whereas a negative value indicates that the median intake in the low education group is higher.

**Figure 5.**
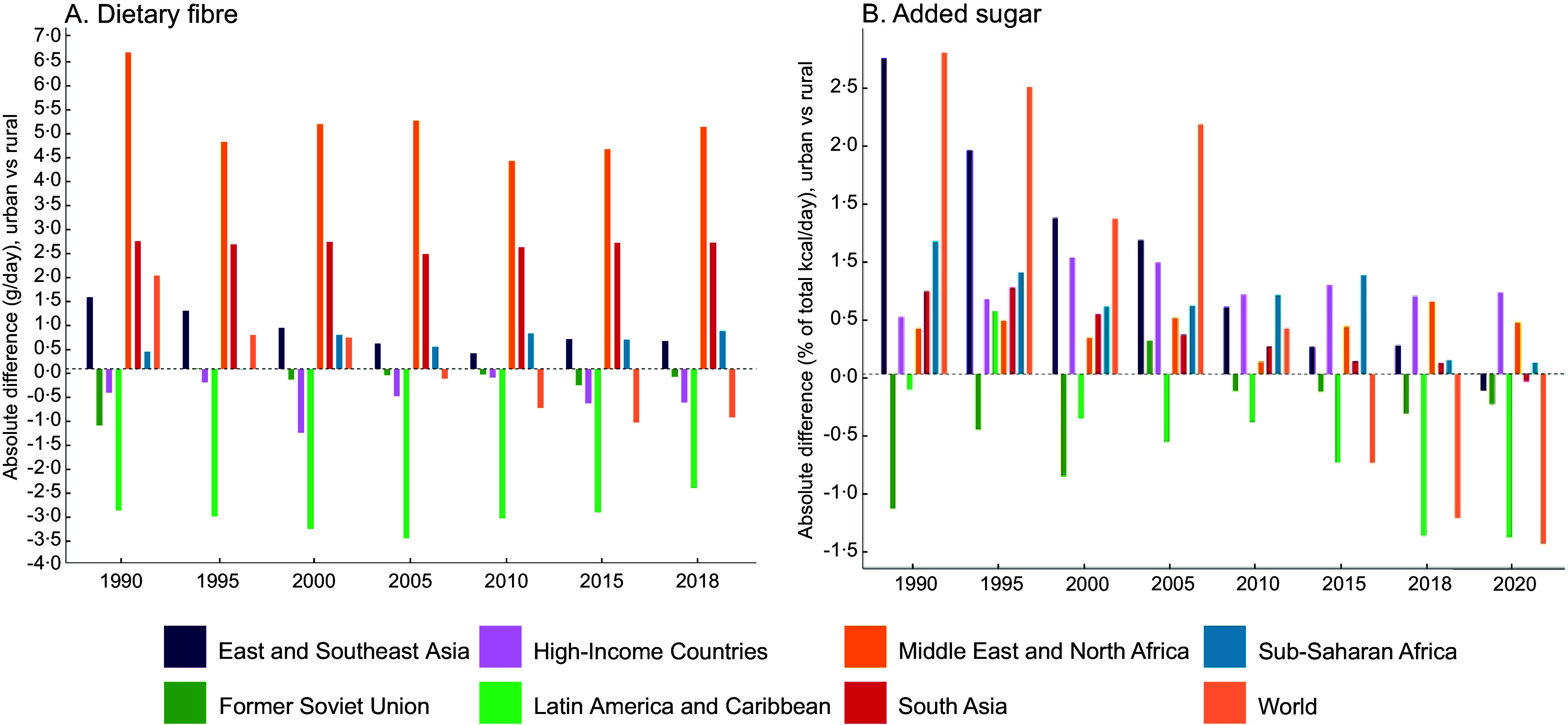
Figure 5 long description. Median difference in absolute dietary intake by residence level and region. (a) Dietary fibre intake (1990–2018). (b) Added sugar intake (1990–2020). A positive value indicates that the median intake in urban areas is higher, whereas a negative value indicates that the median intake in rural areas is higher.

In 2018, East and Southeast Asia was the only region showed a high fibre intake among the higher educated (+1·42 g/d). In contrast, all other regions reported higher fibre intake among the lower educated, including the former Soviet Union (–3·08 g/d), high-income countries (–2·04 g/d), Latin America and the Caribbean (–1·64 g/d), South Asia (–0·91 g/d), the Middle East and North Africa (–0·21 g/d) and Sub-Saharan Africa (–0·04 g/d) (Figure 4(a)). For the effect of urbanicity, higher fibre consumption in urban areas was observed in the Middle East & North Africa (+5·04 g/d), South Asia (+2·63 g/d), East & Southeast Asia (+0·58 g/d) and Sub-Saharan Africa (+0·79 g/d). Conversely, non-urban consumption was higher in high-income countries (–0·71 g/d), Latin America & the Caribbean (–2·48 g/d) and the former Soviet Union (–0·18 g/d) (Figure 5(a)).

In 2020, higher added sugar intake was seen in higher educated populations in high-income countries (+1·99 % of total kcal/d), the Middle East and North Africa (+0·29 % of total kcal/d) and Sub-Saharan Africa (+0·58 % of total kcal/d). However, in all other regions, the lower educated populations continued to show higher intakes, including East and Southeast Asia (–0·61 % of total kcal/d), the former Soviet Union (–0·20 % of total kcal/d), Latin America and the Caribbean (–0·16 % of total kcal/d) and South Asia (–2·6 % of total kcal/d) (Figure 4(b)). For the effect of urbanicity, higher added sugar in urban areas was observed in the Middle East & North Africa (+0·44 % of total kcal/d), high-income countries (+0·7 % of total kcal/d) and Sub-Saharan Africa (+0·09 % of total kcal/d). Conversely, non-urban intake was higher in South Asia (–0·06 % of total kcal/d), East & Southeast Asia (–0·14 % of total kcal/d), Latin America & the Caribbean (–1·4 % of total kcal/d) and the former Soviet Union (–0·26 % of total kcal/d) (Figure 5(b)).

### Dietary fibre and added sugar intake and socio-demographic index

**Figure 6.**
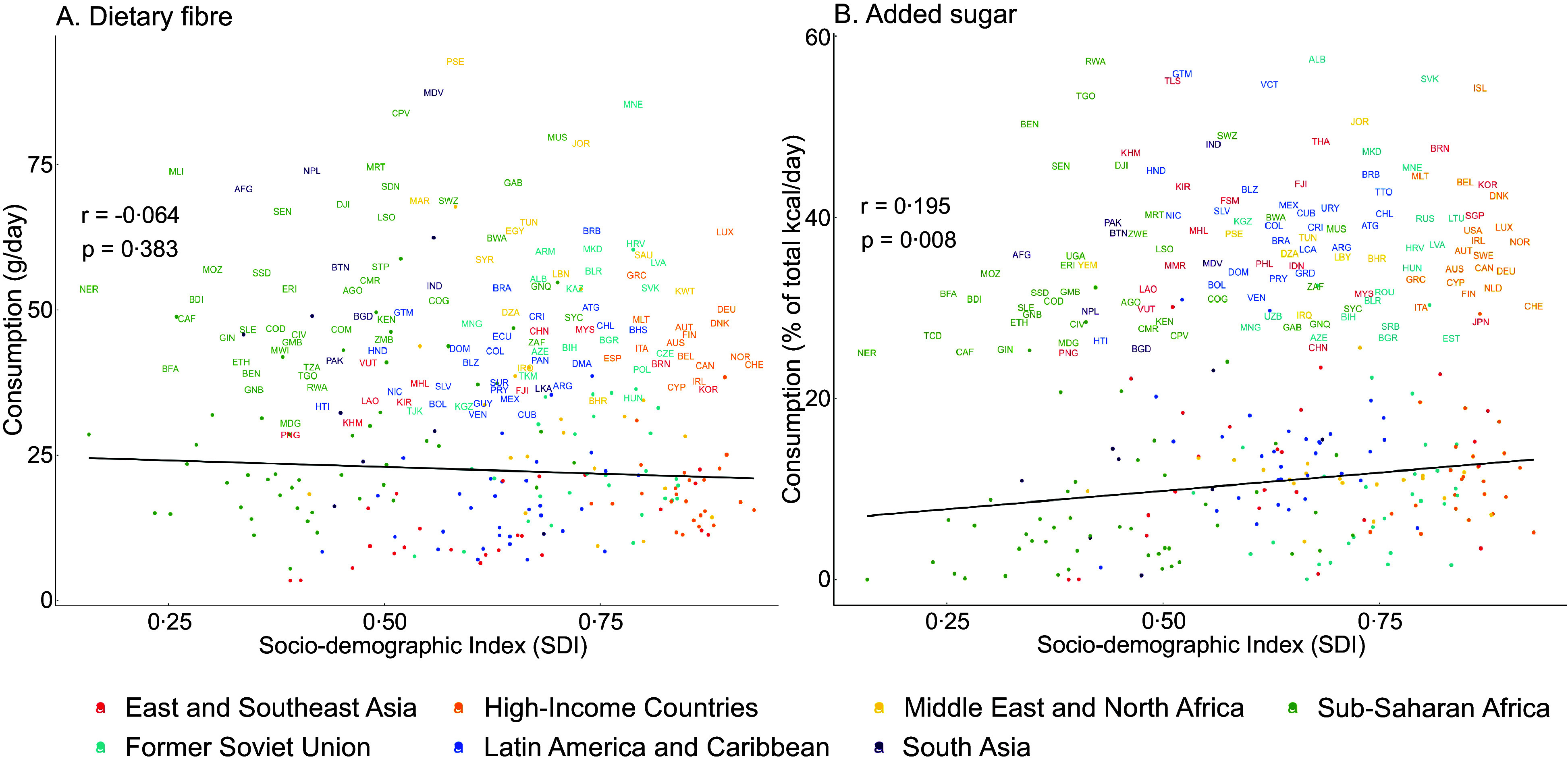
Figure 6 long description. Pearson correlation between (a) dietary fibre intake and (b) added sugar intake and the socio-demographic development index at the national level, stratified by world region.

A correlation analysis was conducted to examine the relationship between national SDI and dietary intake. No significant association was found between national SDI and dietary fibre intake in 2018 (*r* = 0·064, *P* = 0·383). However, a statistically significant positive correlation was observed between added sugar intake and SDI in 2020 (*r* = 0·195, *P* = 0·008) (Figure 6(a) and (b)).

### Association between dietary fibre and added sugar intake and the prevalence of non-communicable diseases

We assessed the associations of dietary fibre intake, added sugar intake and the ratio of dietary fibre intake to added sugar intake with NCD. Dietary fibre intake was positively associated with NCD in both models (Model 1: *β* = 2·81, 95 % CI: 0·97, 4·64, *P* = 0·003; model 2: *β* = 2·40, 95 % CI: 0·65, 4·15, *P* = 0·007), indicating that a one-unit increase in dietary fibre intake was associated with an increase of approximately 2–3 NCD cases per 100 000 population. Added sugar intake was not significantly associated with NCD. The ratio of dietary fibre-to-added-sugar showed an inverse but non-significant association trend with NCD (Model 2: *β* = –10·15, 95 % CI: –21·00, 0·70, *P* = 0·067) (Table 1). Full model results, including coefficients for all covariates, are presented in online supplementary material, Supplemental Table S3.

**Table 1.** Associations of dietary fibre, added sugar and fibre to sugar ratio with NCD prevalence (cases per 100 000 population) across 185 countries (1990–2018) Table 1 long description.

	Model 1			Model 2		
Dietary variables	Coefficient	95 % CI	*P*-value	Coefficient	95 % CI	*P*
Dietary fibre intake (g/d)	2·81	0·97, 4·64	0·003	2·40	0·65, 4·15	0·007
Added sugar (% of total kcal/d)	0·45	−2·20, 3·11	0·739	0·84	–1·69, 3·35	0·516
Dietary fibre to added sugar ratio	−11·32	−22·75, 0·11	0·052	–10·15	–21·00, 0·70	0·067

Results are presented as coefficients (95 % CI), with a significance level set at *P* < 0·05.

Model 1 was adjusted for year and SDI. Model 2 was further adjusted for school enrollment (tertiary, % gross), gross national income (GNI) and rural population (% of total population).

## Discussion

This study utilised data from the GDD, which provides estimates of fibre intake (1990–2018) and added sugar intake (1990–2020) at global, regional and national levels. Over the past three decades, global dietary fibre trends have risen, a finding consistent with the report by Tao, J. *et al.*, likely due to increased public awareness of the benefits of a healthy, high-fibre diet^(9)^. In contrast, global added sugar trends have continued to rise despite public health strategies such as limiting sugary foods in public spaces, taxing sugary products, public awareness campaigns, parental advice and school-based nutrition and restricting related advertising^(24)^. This trend could be attributable to the ongoing nutrition transition^(11)^ and rising consumption of sugar-sweetened beverages^(25)^, posing substantial risks to health and challenges for public health initiatives.

From 1990 to 2019, it was reported that a low-fibre diet contributed to an increased disease burden in southern and central sub-Saharan Africa, as well as increased age-standardised years lived with disability rates in several GBD regions^(26)^. During the same period, high consumption of sugar-sweetened beverages further increased the global disease burden^(27)^. The observed rises in disease burden emphasise the need for additional efforts to reduce the health impacts of low-fibre diet^(26)^ and high sugar intake^(24)^.

In this study, dietary fibre intake decreased only in high-income countries, likely reflecting the high availability and consumption of ultra-processed foods in these regions, which are known to be fibre deficient^(28)^. The increasing trend in added sugar in South Asia may be due to rapid urbanisation and limited regulatory oversight, making the region a primary target for high-sugar packaged goods. In contrast, in Latin America and the Caribbean, the trend is reversing, likely due to strong government regulations promoting a shift to alternative sweeteners^(11)^. At the national level, attention should be drawn to the need for nutrition education programs designed to raise awareness of healthy diets and improve overall health in countries with the lowest median dietary fibre intake and the highest median added sugar intake.

The age-related decline in dietary fibre intake has been previously documented^(29)^. This pattern was also observed in the Middle East and North Africa, the Former Soviet Union and Latin America and the Caribbean and may be explained by age-related health changes as well as socioeconomic factors, including limited social support, lower income and reduced nutrition knowledge^(30)^. Conversely, added sugar intake is highest among younger adults, consistent with prior research^(31)^. However, this pattern was reversed in South Asia and the Former Soviet Union, with higher added sugar intake among older adults, likely reflecting region-specific dietary habits rather than age alone, including higher consumption of processed foods^(32)^ and sugar-sweetened beverages^(25)^. These findings highlight the need for targeted nutrition interventions for older populations in these regions.

This study highlights the influence of socio-demographic factors, particularly education and urbanisation, on dietary fibre and added sugar intake. A cross-sectional study of 6010 participants across ten countries reported that higher education levels correlated with greater dietary fibre knowledge^(33)^. However, lower-educated populations in our study generally reported higher fibre intake. Higher added sugar intake was observed among more educated populations in high-income countries, the Middle East and North Africa and Sub-Saharan Africa. These findings reinforce the idea that nutritional knowledge does not necessarily translate into healthier choices. Previous research supports this, demonstrating a link between higher education and increased fast food consumption rather than fibre-rich whole foods^(34)^.

Studies on dietary intake in rural *v*. urban populations have reported mixed findings, with some indicating higher fibre and added sugar intake in rural areas^(35,36)^ and others in urban populations^(37,38)^, consistent with our findings. In some regions, urban populations consume more dietary fibre and added sugar, likely due to greater access to a wider variety of foods, including both healthier options and less healthy processed products^(39)^. In contrast, in other regions, rural populations may have higher intake, may reflect either traditional fibre-rich diets or the increasing penetration of sugar-rich foods and beverages into rural markets^(36)^. Notably, national SDI was not significantly associated with dietary fibre intake (*P* = 0·383) but was positively correlated with added sugar intake (*P* = 0·008), indicating that higher SDI countries consume more added sugars. These findings suggest that higher-SDI countries should prioritise reducing added sugar intake through taxation and public health campaigns.

Dietary fibre improves cardiometabolic health and reduces NCD risk by optimising glycaemic control (reducing fasting glucose, HbA1c and HOMA-IR), enhancing lipid profiles and lowering blood pressure and systemic inflammation. These effects are mediated by increased intestinal viscosity, which slows glucose absorption, and the fermentation of fibre by gut microbiota into SCFA. SCFA play a pivotal role in regulating insulin sensitivity, lipid metabolism and blood pressure. In addition, dietary fibre promotes satiety through the release of gut hormones such as glucagon-like peptide-1 and cholecystokinin, delays gastric emptying and reduces overall energy absorption^(40,41)^. In contrast, a high intake of added sugars adversely affects cardiometabolic health and increases the risk of NCD by elevating fasting glucose and insulin levels, impairing insulin sensitivity and cellular insulin binding and promoting visceral adiposity. Even short-term consumption increases triglycerides and uric acid, lowers HDL-cholesterol and alters platelet function^(42,43)^. Taken together, these mechanisms provide a biological basis for the observed associations between dietary fibre, added sugar intake and NCD risk.

Studies examining the link between total dietary fibre intake and NCD have yielded inconsistent results^(40,44–47)^, highlighting the complexity of this relationship. Contrary to some previous research, our study identified a positive association between dietary fibre intake and NCD prevalence. In model 1, a 1 g/d increase in dietary fibre intake was associated with a 2·81-unit increase in NCD prevalence (95 % CI: 0·97, 4·64, *P* = 0·003), whereas in model 2, after further adjustment, a 1 g/d increase in dietary fibre intake is associated with a 2·40-unit increase in NCD prevalence (95 % CI: 0·65, 4·15, *P* = 0·007). This unexpected finding warrants further investigation. Despite our attempts to control for relevant factors, residual confounding may have been influencing these results. Specifically, overall diet quality variations, as well as the specific types and quantities of fibre consumed, the presence of pre-existing conditions and lifestyle factors (e.g. exercise, smoking habits and alcohol intake) could play a role in moderating the association between dietary fibre intake and NCD prevalence.

The present analysis did not demonstrate a statistically significant association between the dietary fibre-to-added-sugar ratio and NCD prevalence. The point estimates were in the inverse direction, which is consistent with the established beneficial effects of dietary fibre and the adverse effects of added sugar^(48,49)^. However, given the lack of statistical significance, these findings should be considered hypothesis generating rather than conclusive. Several limitations inherent to ecological studies may explain the absence of statistical significance, including the ecological fallacy, residual country-level confounding (e.g. healthcare access) and measurement error in country-level dietary estimates. Future individual-level prospective studies are needed to properly test this hypothesis.

This study has certain limitations that warrant consideration. Notably, it is crucial to consider the inherent limitations arising from the ecological fallacy of this study, meaning that observed associations at the aggregate level might not truly represent relationships at the individual level. Consequently, our findings may not be generalisable to individual dietary habits and NCD risks. Additionally, the availability of dietary intake data at only 5-year intervals and the limited data for certain countries may affect the precision of our estimates. The absence of total energy data in the GDD prevented conversion of added sugar from % kcal/d to grams, necessitating the use of a fibre-to-added-sugar ratio, which may not fully capture the complex interplay between these dietary components. Furthermore, GDD estimates showed uncertainty: mean 95 % CI width exceeded temporal sd for 88·1 % (dietary fibre) and 96·2 % (added sugar) of countries. Excluding the nineteen highest-uncertainty countries per nutrient (10·3 %) yielded consistent LMM results, suggesting findings are robust to imprecise estimates (online supplementary material, Supplemental Figure S4; Table S4). Model diagnostics indicated mild heteroscedasticity and non-normal tails, which are typical of large cross-country data. We acknowledge that SDI, GNI and school enrollment capture conceptually related dimensions of socio-economic development. However, generalised variance inflation factors ( $GVIF^{1/\left(2\times Df\right)})$ were all below 2·5 (range: 1·04–2·34), indicating no problematic multicollinearity.

This study has several strengths. To our knowledge, it is the first to report dietary fibre and added sugar intake stratified by age, education level and urbanicity, covering global, regional and national populations. Our research utilises multiple high-quality data sources, including the GDD, the GBD and the World Development Indicators. This approach offers a comprehensive overview of dietary trends and their association with NCDs over the past three decades. By employing a longitudinal perspective, we can examine temporal changes in dietary intake at the country level, providing a more nuanced understanding of trends compared with cross-sectional analyses. Our models weighted countries equally, regardless of population size. This ensures that small countries are not overshadowed by populous nations.

Future research is needed to examine fibre subtypes or fibre from different sources to enhance mechanistic understanding and guide precise recommendations. Disaggregating NCD outcomes by specific condition will clarify whether the fibre-to-added-sugar ratio differentially affects CVD, diabetes or cancers. Individual-level prospective studies are needed to establish causality and exclude reverse causation.

### Conclusion

This study reveals significant global variations in dietary fibre and added sugar consumption, highlighting substantial differences across regions and populations. Our analysis documents trends over time, quantifies national intake levels and explores the associations between these dietary components and NCD. Our study may suggest a potential relevance of the fibre-to-added-sugar ratio to NCD prevalence. These findings underscore the need for tailored national policies and targeted interventions to promote increased fibre intake, particularly in high-income countries, and to curb excessive added sugar consumption, especially in South Asia. This evidence base can inform the design of more effective strategies to improve global dietary habits and mitigate the burden of NCD.

## Supporting information

10.1017/S1368980026103188.sm001 Alballa et al. supplementary material Alballa et al. supplementary material

## Data Availability

For this study, we downloaded estimates of dietary fibre intake and added sugar intake from the Global Dietary Database (GDD) dataset available through the Global Health Data Exchange (GHDx) (https://ghdx.healthdata.org/organizations/global-dietary-database). Age-specific population weights were obtained from the United Nations Population Division (https://population.un.org/wpp/). The prevalence rate data for NCD were sourced from the GBD website (https://ghdx.healthdata.org/gbd-results-tool). The SDI data were also obtained from the GBD (https://ghdx.healthdata.org/record/ihme-data/gbd-2019-socio-demographic-index-sdi-1950-2019). Furthermore, covariate data were sourced from the WDI website (https://data.worldbank.org/).

## Supplementary material

For supplementary material accompanying this paper visit https://doi.org/10.1017/S1368980026103188

## Figure 1. Long description

Panel A, titled “Dietary Fiber” shows consumption in grams per day from 1990 to 2018. Panel B, titled “Added sugar” shows consumption as a percentage of total kcal/day from 1990 to 2020. Eight colored lines represent the global intake and different regions: East & Southeast Asia, Former Soviet Union, High-Income Countries, Latin America & Caribbean, South Asia, Middle East & North Africa, and Sub-Saharan Africa. Globally, dietary fiber and added sugar intake have increased over the years. Regionally, dietary fiber intake increased in all regions between 1990 and 2018, except in high-income countries. The Middle East and North Africa had the highest fiber intake in 2018, followed by South Asia and Sub-Saharan Africa (Fig. 1A). Regarding added sugar, all regions showed an increase in intake between 1990 and 2020, except for Latin America & Caribbean. South Asia reported the highest added sugar intake in 2020, while East and Southeast Asia had the lowest (Fig. 1B).

Navigate back to Figure 1..

## Figure 2. Long description

Panel A, titled “Dietary fiber” shows a world map color-coded by national median dietary fiber consumption in grams per day in 2018. The color scale ranges from light yellow (3.45–9.92 g/day) to dark brown (61.31–67.72 g/day). Panel B, titled “Added sugar” shows a world map color-coded by national median added sugar consumption as a percentage of total kcal per day in 2020. The color scale ranges from very light purple (0.00–3.24%) to dark purple (29.22–32.46%). Countries are shaded according to their respective consumption levels in each panel.

Navigate back to Figure 2..

## Figure 3. Long description

Panel A shows dietary fiber consumption (g/day) by age (0.5–96.5 years) in 2018. Middle East and North Africa has the highest intake across almost all ages. East and Southeast Asia, High-Income Countries, and Sub-Saharan Africa show increased intake with age. Middle East and North Africa, Former Soviet Union, and Latin America and Caribbean show decreased intake with age. South Asia shows minimal variation between adults and elderly. Panel B shows added sugar consumption (% of total kcal/day) by age in 2020. South Asia has the highest intake across all ages. Former Soviet Union shows slightly higher intake in aged 95+ versus adults. All other regions show decreased added sugar intake among older individuals compared to younger adults.

Navigate back to Figure 3..

## Figure 4. Long description

Panel A shows absolute differences in dietary fiber (g/day) between high and low education levels from 1990 to 2018. Positive values indicate higher intake in high-education groups; negative values indicate higher intake in low-education groups. Most bars are negative, showing low-education groups generally consume more fiber. In 2018, only East and Southeast Asia showed higher fiber intake among the higher educated. All other regions reported higher fiber intake among the lower educated. Panel B shows absolute differences in added sugar (% of total kcal/day) between high and low education levels from 1990 to 2020. Most bars are positive, indicating higher added sugar intake among high-education groups. In 2020, higher added sugar intake was seen in higher educated populations in High-Income Countries, Middle East and North Africa, and Sub-Saharan Africa. In all other regions, lower educated populations showed higher intakes.

Navigate back to Figure 4..

## Figure 5. Long description

Panel A shows absolute differences in dietary fiber (g/day) between urban and rural areas from 1990 to 2018. Positive values indicate higher urban intake; negative values indicate higher rural intake. East & Southeast Asia, Middle East & North Africa, and South Asia show positive differences, indicating higher fiber intake in urban areas. Latin America & Caribbean shows negative differences, indicating higher fiber intake in rural areas. Panel B shows absolute differences in added sugar (% of total kcal/day) between urban and rural areas from 1990 to 2020. East & Southeast Asia, Middle East & North Africa, High-Income Countries, and Sub-Saharan Africa show positive differences in most of the years, indicating higher added sugar intake in urban areas. Former Soviet Union and Latin America & Caribbean show negative differences, indicating higher added sugar intake in rural areas.

Navigate back to Figure 5..

## Figure 6. Long description

Panel A, titled “Dietary fiber” shows the relationship between dietary fiber consumption (g/day) and the Socio-demographic Index (SDI). Each colored dot represents a country, stratified by world region. The correlation coefficient is r = -0.064 with p = 0.383, indicating no significant relationship. Panel B, titled “Added sugar” shows the relationship between added sugar consumption (% of total kcal/day) and SDI. The correlation coefficient is r = 0.195 with p = 0.008, a statistically significant positive relationship indicating that higher SDI countries consume more added sugars.

Navigate back to Figure 6..

## Table 1. Long description

Dietary fiber intake was positively associated with NCDs in both models (Model 1: β = 2.81, 95% CI: 0.97-4.64, p = 0.003; Model 2: β = 2.40, 95% CI: 0.65-4.15, p = 0.007), indicating that a one-unit increase in dietary fiber intake was associated with an increase of approximately 2–3 NCD cases per 100,000 population. Added sugar intake was not significantly associated with NCDs. The ratio of dietary fiber-to-added-sugar showed an inverse but non-significant association trend with NCDs (Model 2: β = −10.15, 95% CI: −21.00 to 0.70, p = 0.067).

Navigate back to Table 1..
