# Supplementary material for: Variations in dietary fibre and added sugar intake across global, regional and national levels: implications for non-communicable diseases
Source: Public Health Nutr. 2026 Jul 21;29(1):e169. doi: 10.1017/S1368980026103188 (PMC13469227; doi:10.1017/S1368980026103188)
Supplement: Supplementary file 1 [file S1368980026103188sup001.docx]

**Supplementary Figure S1** Diagnostic plots for the dietary fiber linear mixed effects model

**Supplementary Figure S2** Diagnostic plots for the added sugar linear mixed effects model

**Supplementary Figure S3** Diagnostic plots for the fiber to added sugar ratio linear mixed effects model

**Supplementary Figure S4** Country level dietary fiber and added sugar trends highlighting high-uncertainty estimates

**Supplementary Table S1.** GDD variables definition

| **GDD Variable Label** | **GDD Variable Unit** | **GDD Definition** |
| --- | --- | --- |
| **Dietary fiber** | grams per day | Total dietary fiber intake from all sources (fruits, vegetables, grains, legumes, pulses), defined as the carbohydrate polymers which are not hydrolyzed by the endogenous enzymes in the small intestine of human being. |
| **Added sugars** | % of total kcal per day (energy contribution) | Total intake of sugars added during the preparation or processing of foods and beverages. Examples include the sugars added in sugar-sweetened beverages, desserts, candy, breakfast cereals, and sweetened milk. This definition excludes non-caloric sweeteners and sugars that naturally occur in food, such as those in fruits, milk or milk products. |

**Supplementary Table S2:** GDD Geographic Regions

| **Asia** | **Asia East:** China, Taiwan.  **Asia-Pacific High-Income**: Brunei Darussalam, Japan, Republic of Korea, Singapore.  **Asia, Southeast:** Cambodia, Indonesia, Lao People's Democratic Republic, Malaysia, Myanmar, Philippines, Thailand, Timor-Leste, Viet Nam.  **Oceania**: Fiji, Kiribati, Marshall Islands, Micronesia, Papua New Guinea, Samoa, Solomon Islands, Tonga, Vanuatu. |
| --- | --- |
| **Former Soviet Union** | **Asia, Central;** Armenia, Azerbaijan, Georgia, Kazakhstan, Kyrgyzstan, Mongolia, Tajikistan, Turkmenistan, Uzbekistan.  **Europe, Central:** Albania, Bosnia and Herzegovina, Bulgaria, Croatia, Czechia, Hungary, Montenegro, Poland, Romania, Serbia, Slovakia, Slovenia, The former Yugoslav Republic of Macedonia.  **Europe, Eastern:** Belarus, Estonia, Latvia, Lithuania, Republic of Moldova, Russian Federation, Ukraine. |
| **Latin America and Caribbean** | **Caribbean:** Antigua and Barbuda, Bahamas, Belize, Barbados, Cuba, Dominica, Dominican Republic, Grenada, Guyana, Haiti, Jamaica, Saint Lucia, Saint Vincent and the Grenadines, Suriname, Trinidad and Tobago.  **Latin America, Andean:** Bolivia (Plurinational State of), Ecuador, Peru.  **Latin America, Southern**: Argentina, Chile, Uruguay.  **Latin America, Tropical**: Brazil, Paraguay.  **Latin America, Central:** Colombia, Costa Rica, El Salvador, Guatemala, Honduras, Mexico, Nicaragua, Panama, Venezuela (Bolivarian Republic of). |
| **Middle East and North Africa** | **Europe, Western:** Israel  **North Africa and Middle East:** Algeria, Bahrain, Egypt, Iran (Islamic Republic of), Iraq, Jordan, Kuwait, Lebanon, Morocco, Occupied Palestinian Territory, Oman, Qatar, Saudi Arabia, Syrian Arab Republic, Tunisia, Turkey, United Arab Emirates, Yemen. |
| **South Asia** | **Asia, South:** Afghanistan, Bangladesh, Bhutan, India, Nepal, Pakistan.  **Asia, Southeast:** Maldives, Sri Lanka. |
| **Sub-Saharan Africa** | **Sub-Saharan Africa, Central:** Angola, Central African Republic, Congo, Democratic Republic of the Congo, Equatorial Guinea, Gabon.  **Sub-Saharan Africa, Southern**: Botswana, Lesotho, Namibia, South Africa, Swaziland, Zimbabwe.  **Sub-Saharan Africa, East:** Burundi, Comoros, Djibouti, Eritrea, Ethiopia, Kenya, Madagascar, Malawi, Mauritius, Mozambique, Rwanda, Seychelles, South Sudan, Sudan, Uganda, United Republic of Tanzania, Zambia.  **Sub-Saharan Africa, West:** Benin, Burkina Faso, Cameroon, Cape Verde, Chad, Côte d'Ivoire, Gambia, Ghana, Guinea, Guinea-Bissau, Liberia, Mali, Mauritania, Niger, Nigeria, São Tomé and Príncipe, Senegal, Sierra Leone, Togo. |
| **High-Income Countries** | **Australasia:** Australia, New Zealand.  **Europe, Western:** Austria, Belgium, Cyprus, Denmark, Finland, France, Germany, Greece, Iceland, Ireland, Italy, Luxembourg, Malta, Netherlands, Norway, Portugal, Spain, Sweden, Switzerland, United Kingdom.  **North America High-Income:** Canada, United States of America. |

**Supplementary Table S3:** Associations of dietary fiber, added sugar, and fiber to added sugar ratio with NCD prevalence (cases per 100,000 population) across 185 countries (1990-2018)

|  | **Model 1** | |  | **Model 2** | |
| --- | --- | --- | --- | --- | --- |
|  | **Coefficient (95%CI)** | ***P*-value** |  | **Coefficient (95%CI)** | ***P-value*** |
|  |  |  |  |  |  |
| **Dietary fiber intake (g/day)** | 2.81 (0.93) | 0.003 |  | 2.40 (0.89) | 0.007 |
| **Year 1990 (ref)** |  |  |  |  |  |
| **Year 1995** | 70.64 (20.68) | <0.001 |  | 62.89 (19.66) | 0.001 |
| **Year 2000** | 137.98 (22.23) | <0.001 |  | 116.59 (21.32) | <0.001 |
| **Year 2005** | 204.50 (24.76) | <0.001 |  | 160.96 (24.13) | <0.001 |
| **Year 2010** | 257.72 (28.06) | <0.001 |  | 195.00 (27.68) | <0.001 |
| **Year 2015** | 366.46 (31.66) | <0.001 |  | 288.87 (31.46) | <0.001 |
| **Year 2018** | 449.23 (33.76) | <0.001 |  | 365.55 (33.55) | <0.001 |
| **SDI** | -254.31 (171.56) | 0.139 |  | -72.88 (36.68) | 0.047 |
| **GNI** |  |  |  | 63.70 (10.78) | <0.001 |
| **Rural population** |  |  |  | 41.73 (34.10) | 0.221 |
| **School enrollment** |  |  |  | 126.03 (14.58) | <0.001 |
| **AIC** | 18169.58 |  |  | 18035.09 |  |
|  |  |  |  |  |  |
| **Added sugar (% of total kcal/day)** | 0.45 (1.35) | 0.739 |  | 0.84 (1.28) | 0.516 |
| **Year 1990 (ref)** |  |  |  |  |  |
| **Year 1995** | 70.59 (20.73) | <0.001 |  | 62.86 (19.70) | 0.001 |
| **Year 2000** | 140.24 (22.29) | <0.001 |  | 118.27 (21.37) | <0.001 |
| **Year 2005** | 208.89 (24.80) | <0.001 |  | 164.06 (24.17) | <0.001 |
| **Year 2010** | 263.43 (28.11) | <0.001 |  | 198.77 (27.73) | <0.001 |
| **Year 2015** | 374.18 (31.70) | <0.001 |  | 294.05 (31.51) | <0.001 |
| **Year 2018** | 455.87 (33.85) | <0.001 |  | 369.63 (33.64) | <0.001 |
| **SDI** | -234.08 (173.06) | 0.177 |  | -73.32 (36.96) | 0.048 |
| **GNI** |  |  |  | 63.96 (10.81) | <0.001 |
| **Rural population** |  |  |  | 36.67 (34.15) | 0.283 |
| **School enrollment** |  |  |  | 127.74 (14.59) | <0.001 |
| **AIC** | 18177.69 |  |  | 18041.13 |  |
|  |  |  |  |  |  |
| **Dietary fiber to added sugars ratio** | -11.32 (5.83) | 0.052 |  | -10.15 (5.53) | 0.067 |
| **Year 1990 (ref)** |  |  |  |  |  |
| **Year 1995** | 70.78 (20.70) | <0.001 |  | 62.91 (19.68) | 0.001 |
| **Year 2000** | 140.80 (22.25) | <0.001 |  | 118.72 (21.35) | <0.001 |
| **Year 2005** | 209.84 (24.78) | <0.001 |  | 164.99 (24.16) | <0.001 |
| **Year 2010** | 264.95 (28.08) | <0.001 |  | 200.46 (27.71) | <0.001 |
| **Year 2015** | 376.12 (31.67) | <0.001 |  | 296.24 (31.48) | <0.001 |
| **Year 2018** | 460.27 (33.85) | <0.001 |  | 374.13 (33.65) | <0.001 |
| **SDI** | -46.11 (33.11) | 0.164 |  | -72.11 (36.77) | 0.050 |
| **GNI** |  |  |  | 63.56 (10.78) | <0.001 |
| **Rural population** |  |  |  | 38.27 (34.13) | 0.262 |
| **School enrollment** |  |  |  | 127.29 (14.57) | <0.001 |
| **AIC** | 18174.41 |  |  | 18035.28 |  |
|  |  |  |  |  |  |

Results are presented as β (SE), with a significance level set at P < 0.05.

Model 1 was adjusted for year and SDI as fixed effects. Model 2 further added GNI, rural population, and school enrollment as fixed effects. In both models, country was included as a random effect.

**Supplementary Table S4:** Linear mixed effects model for added sugar and dietary fiber intake and NCD prevalence after excluding countries with high uncertainty in GDD estimates

|  |  | **Model 1** | |  | **Model 2** | |
| --- | --- | --- | --- | --- | --- | --- |
| **Dietary variables** | **N of countries** | **Coefficient (95%CI)** | ***P*-value** |  | **Coefficient (95%CI)** | ***P-value*** |
|  |  |  |  |  |  |  |
| **Dietary fiber intake (g/day)** | 166 | 4.65 (2.66 – 6.65) | <0.001 |  | 3.91 (1.99 – 5.84) | <0.001 |
|  |  |  |  |  |  |  |
| **Added sugar intake**  **(% of total kcal/day)** | 166 | -0.92 (-3.68 – 1.83) | 0.511 |  | -0.54 (-3.22 – 2.13) | 0.691 |
|  |  |  |  |  |  |  |

Results are presented as coefficients (95% Confidence Interval), with a significance level set at P < 0.05.

Countries with high uncertainty in GDD estimates were excluded separately for each nutrient (19 countries for dietary fiber; 19 for added sugar; with 8 countries overlapping between the two sets).

Model 1 was adjusted for year and SDI as fixed effects. Model 2 further added GNI, rural population, and school enrollment as fixed effects. In both models, country was included as a random effect.
